# Supplementary material for: Patterns of Intron Gain and Loss in Fungi
Source: PLoS Biol. 2004 Nov 30;2(12):e422. doi: 10.1371/journal.pbio.0020422 (PMC532390; doi:10.1371/journal.pbio.0020422)
Supplement: Table S1 — Also available at http://genes.mit.edu/NielsenEtAl/. (4.3 MB ZIP). [file pbio.0020422.st001.zip › NielsenEtAl/html/1372.html]

AN2414.1.NCU02534.1.MG09285.1.FG00376.1


```
 CLUSTAL W (1.82) Multiple Sequence Alignments - Introns Inserted


Sequence 1: NCU02534.1	492 aa
Sequence 2: FG00376.1	468 aa
Sequence 3: MG09285.1	488 aa
Sequence 4: AN2414.1	468 aa
Alignment Length: 506 aa
Number Identitical Residues: 344 aa
Alignment Score (without introns) 14581


MG09285.1 	MAS-LYRLAGRGASKRLCLRPATSSQIPISASCSSAASTIRSLSSTTRRRYATTPDPAEL
NCU02534.1	MATTLFRLAGR-NAKRHCMRQST--------TIAHNLNSTRAFSASALRRYAEPS-----
FG00376.1 	MAT--LRLASR-GVKSLCMRPAT--------FAAR------PFSTTCLRRNAAP------
AN2414.1  	-------MAGN-APKKLCLRPSG-------FARSTLVPRSRSIATSVSRRATEPT-----
          	       :*..   *  *:* :           :       .::::  ** : ..     

MG09285.1 	IKAQATRLVPVDSDFSNPVDPYFVTNRKDDIEANEPG---VAERKIRHYTVNFGPQHPAA
NCU02534.1	YEGQGTRLVPTGDDFAPNNDLYGLEALKADGAPRVPPQDHILARKVRHYTVNFGPQHPAA
FG00376.1 	VETVGTRLVPVDEDFSSAQDSYGLSKPHTPGT-RKSRENSVSDRKVRHYTVNFGPQHPAA
AN2414.1  	-SYQATRLIPTDPTFSHFINKDSDVPETSAGLESEAEG---VGRKIRHYTVNFGPQHPAA
          	 .  .***:*..  *:   :               .       **:**************

MG09285.1 	HGVLRLILELSGEEIVRADPHVGLLHRGTEKLCEYKTYLQALPYFDRLDYVSMMTNEQCF
NCU02534.1	HGVLRLILELKGEEIVRADPHVGLLHRGTEKLCEYRTYLQALPYFDRLDYVSMMTNEQCF
FG00376.1 	HGVLRLILELNGEEIVRADPHVGLLHRGTEKLIEYKTYLQALPYFDRLDYVSMMTNEQCF
AN2414.1  	HGVLRLILEINGEEIVRADPHVGLLHRGTEKLIEYKSYFQALPYFDRLDYVSMMTNEQCY
          	*********:.********************* **::*:********************:

MG09285.1 	ALAVEKLLNIEIPERAKYIRTMFGEITRVLNHLMSVLSHAMDVGALTPFLWGFEEREKLM
NCU02534.1	ALAVEKLLNVEIPERAKWIRTMFAEITRILNHLMSVLSHAMDVGALTPFLWGFEEREKLM
FG00376.1 	SLAVEKLLNVEIPERAKWIRTLFGEITRILNHLMSVLSHAMDVGALTPFLWGFEEREKLM
AN2414.1  	SLAVEKLLNIEIPERAKYIRTMFGEITRILNHLMSVLSHAMDVGALTPFLWGFEEREKLM
          	:********:*******:***:*.****:*******************************

MG09285.1 	0EFYERVSGARLHAAYVRPGGVHQDIPAGLLDDIYQWATQFGDRIDETEEMLTDNRIWIN
NCU02534.1	0EFYERVSGARLHAAYVRPGGVHQDIPLGLLDDIYMWATQFGDRIDETEEMLTDNRIWID
FG00376.1 	0EFYERVSGARLHAAYVRPGGVHQDIPVGLLDDIYQWATQFGDRIDETEEMLTDNRIWIE
AN2414.1  	0EFYERVSGARLHAAYVRPGGVSQDLPLGLLDDIYQWATQFGDRIDETEELLTDNRIWKA
          	 ********************* **:* ******* **************:*******  

MG09285.1 	RLQGVGVVSAKEALDLSFTGVMLRGSGVPWDIRKSSPYDAYDKVEFDIPVGLNGDCYDRY
NCU02534.1	RLRGIGVVSAADALNLSFTGVMLRGSGVPWDIRKSQPYDAYDQVEFDVPVGINGDCYDRY
FG00376.1 	RLRGVGVVPAAEALNLSFTGVMLRGSGVPFDVRKNQPYDAYDQVEFDVPVGTNGDCYDRY
AN2414.1  	RTQGVGVVSAADALNMSFTGVMLRGSGVPWDIRKSQPYDAYDKVEFDVPVGVNGDCYDRY
          	* :*:***.* :**::*************:*:**..******:****:*** ********

MG09285.1 	LCRMEEFRQSLRIIHQCLNQMPAGPVRVEDYKVSPPPRAAMKENMEALIHHFLLYTKGYA
NCU02534.1	LCRMEEFRQSLRIIHQCLNKMPAGPVRVEDYKISPPPRSAMKENMEALIHHFLLYTKGYA
FG00376.1 	LCRMEEFRQSLRIIHQCLNKMPAGPVRVEDYKVSPPPRAAMKENMEALIHHFLLYTKGYA
AN2414.1  	LCRMEEFRQSLRIIHQCLNQMPAGPVRVEDYKIMPPPRAAMKENMEALIHHFLLYTKGYA
          	*******************:************: ****:*********************

MG09285.1 	VPPGDTYSVIEAPKGEMGVYVVSDGSERPYRVHIRAPGFAHLAGFDHVCKGHLLADAVAV
NCU02534.1	VPPGDTYSAIEAPKGEMGVYVVSDGSERPYRVHIRAPGFAHLGGFDHLSRGHMLADAVAV
FG00376.1 	VPPGETYSAIEAPKGEMGVYVVSDGSERPYRCHIRAPGFAHLGGFDHVSKGHLLADAVAV
AN2414.1  	VPPGETYSAIEAPKGEMGVFLVSDGSERPYRCKIRAPGFAHLGGFDQIARGHLLADAVAI
          	****:***.**********::********** :*********.***::.:**:******:

MG09285.1 	IG~TMDLVFG1EVDR--------------
NCU02534.1	IG~TMDLVFG~KFDSHCVKLSSLKFQVVW
FG00376.1 	IG~TMDLVFG1EVDR--------------
AN2414.1  	IG1TMDLVFG~EVDR--------------
          	** ******* :.*
```
